# Microscopic colitis: Etiopathology, diagnosis, and rational management

**DOI:** 10.7554/eLife.79397

**Published:** 2022-08-01

**Authors:** Ole Haagen Nielsen, Fernando Fernandez-Banares, Toshiro Sato, Darrell S Pardi

**Affiliations:** 1 Department of Gastroenterology, Herlev Hospital, University of Copenhagen Herlev Denmark; 2 Department of Gastroenterology, Hospital Universitari Mutua Terrassa Barcelona Spain; 3 Centro de Investigación Biomédica en Red de enfermedades hepáticas y digestivas CIBERehd Spain; 4 Department of Gastroenterology, Keio University School of Medicine Tokyo Japan; 5 Division of Gastroenterology and Hepatology, Mayo Clinic Rochester United States; University of Bern Switzerland; Harvard T.H. Chan School of Public Health United States

**Keywords:** Microscopic colitis, collagenous colitis, lymphocytic colitis, etiopathology, therapy, prognosis

## Abstract

Microscopic colitis is an inflammatory bowel disease divided into two subtypes: collagenous colitis and lymphocytic colitis. With an increasing incidence of microscopic colitis exceeding those of ulcerative and Crohn’s disease among elderly people in some countries, microscopic colitis is a debilitating life experience. Therefore, physicians should be familiar with its clinical features and management strategies because the disease deserves the same attention as the classical inflammatory bowel diseases. Here, state-of-the-art knowledge of microscopic colitis is provided from a global perspective with reference to etiopathology and how to establish the diagnosis with the overall aim to create awareness and improve rational management in clinical practice. The immune system and a dysregulated immune response seem to play a key role combined with risk factors (e.g. cigarette smoking) in genetically predisposed individuals. The symptoms are characterized by recurrent or chronic nonbloody, watery diarrhea, urgency, weight loss, and a female preponderance. As biomarkers are absent, the diagnosis relies on colonoscopy with a histological assessment of biopsy specimens from all parts of the colon. Although the disease is not associated with a risk of colorectal cancer, a recent nationwide, population-based cohort study found an increased risk of lymphoma and lung cancer. Budesonide is the first-line therapy for management, whereas immunomodulatory drugs (including biologics) and drugs with antidiarrheal properties may be indicated in those failing, dependent, or intolerant to budesonide. In microscopic colitis induced by checkpoint inhibitors, a drug class used increasingly for a wide range of malignancies, a more aggressive therapeutic approach with biologics introduced early seems reasonable. However, particular attention needs to be drawn to the existence of incomplete forms of microscopic colitis with the risk of being overlooked in routine clinical settings.

## Introduction

Microscopic colitis, of which two major histological subtypes (collagenous colitis and lymphocytic colitis) exist, is a common cause of chronic or recurrent, nonbloody, watery diarrhea. Since the symptoms of microscopic colitis are nonspecific and the diagnosis requires histology, the disease risks being overlooked (Nielsen et al., 2004). It is affiliated to the umbrella diagnosis of inflammatory bowel disease (IBD) (Escudero-Hernández et al., 2021) and is described by a clinicopathological triad characterized by a history of chronic or intermittent watery diarrhea, normal or almost normal endoscopic examination of the colon (e.g., with slight edema, erythema, and/or loss of vascular pattern, although rarely more significant macroscopic changes are reported, including pseudomembranes and ‘cat scratch changes’) (Marlicz et al., 2004), as well as a distinct histological pattern when examined under a microscope – hence the name of this disorder.

The ‘classical’ entities of IBD, Crohn’s disease and ulcerative colitis, are distinct from microscopic colitis in that they cause macroscopic inflammation that is visible endoscopically and/or on cross-sectional imaging. In contrast, microscopic colitis appears largely normal on endoscopic inspection and cross-sectional imaging. In addition, microscopic colitis is distinct from IBD histologically, with the former showing either a prominent intraepithelial lymphocytosis (lymphocytic colitis) or a thickened subepithelial collagen band (collagenous colitis), while the latter (IBD) shows crypt abscesses, crypt architectural distortion (signifying chronic crypt destruction inflammation), and in some cases of Crohn’s disease, granulomas (Vespa et al., 2022). Moreover, ulcerative colitis and Crohn’s disease have their peak incidence in late adolescence and early adulthood (Lophaven et al., 2004).

Although most patients receive their diagnosis at an age of 60 or above, approximately 25% of patients get diagnosed before the age of 45 (Bonderup et al., 2015). The disease has even been reported in children, which, however, is very rare (Windon et al., 2020). A female preponderance exists in both collagenous and lymphocytic colitis, with a female-to-male ratio of 3.1 and 1.9, respectively (Tong et al., 2015).

The objectives of this article are to provide scientific generalists a global view of recent advances in etiopathology and clinical data of microscopic colitis that might improve awareness to reach the correct diagnosis and assist to establish rational management at an early point for the benefit of the patients affected.

## Epidemiology and risk factors

**Figure 1.**
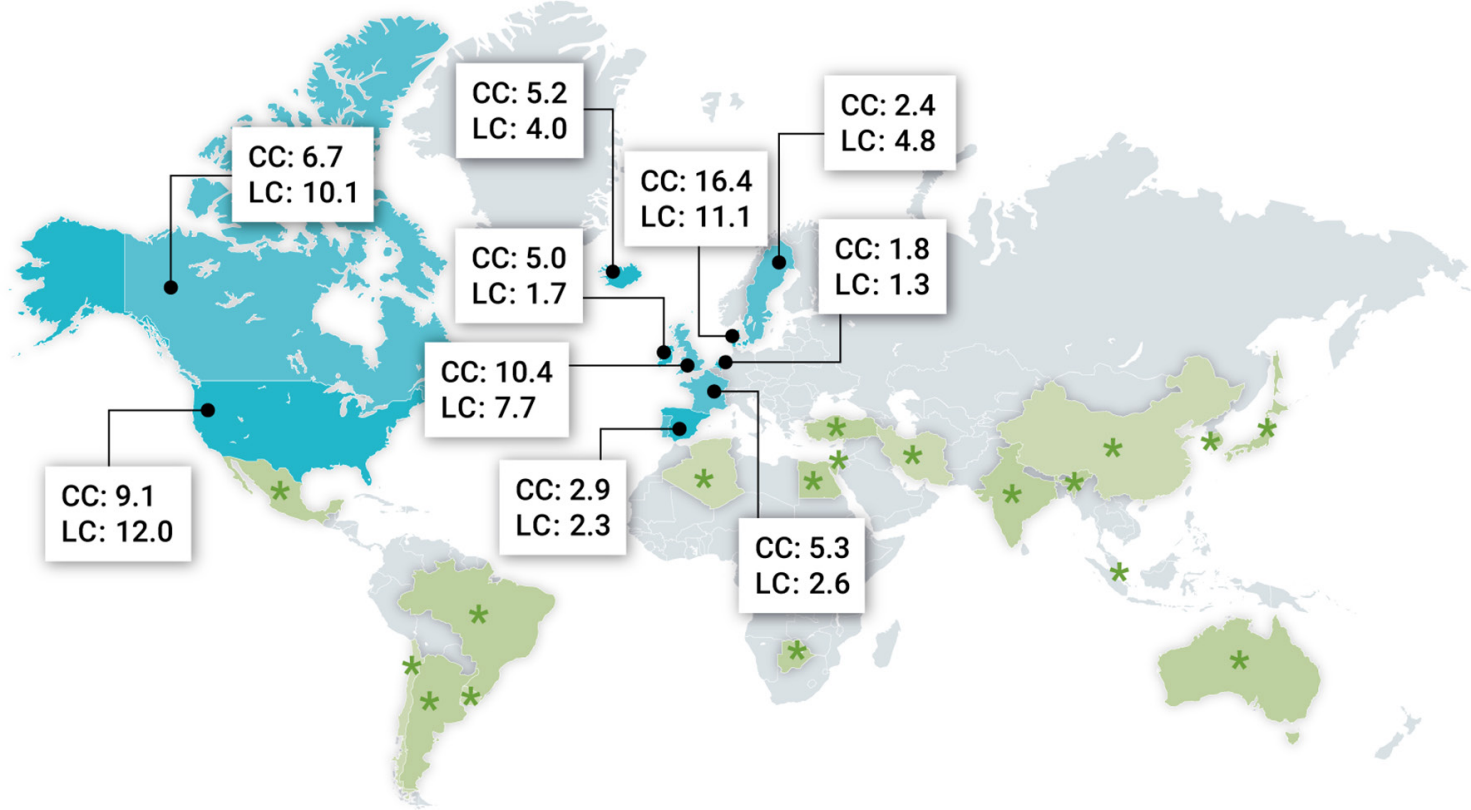
Geographic distribution of microscopic colitis in different parts of the world. Most recent incidence rates (× 10^5^ inhabitants per year) of both collagenous colitis (CC) and lymphocytic colitis (LC) from Europe and North America where population-based studies have been performed. Moreover, green asterisks indicate countries where microscopic colitis has been described outside Europe and North America but without incidence data. Data retrieved from Miehlke et al., 2021; Davidson et al., 2018.

A meta-analysis has revealed a pooled worldwide incidence of microscopic colitis of 4.9 (95% CI 4.2–5.7) cases per 100,000 patient-years for collagenous colitis and 5.0 (95% CI 4.0–6.1) cases per 100,000 patient-years for lymphocytic colitis (Miehlke et al., 2021). Estimates of incidence originate predominantly from North America and Europe (Figure 1), and a substantial variation in incidence among regions has been observed.

Epidemiological studies have shown that in some countries the incidence of microscopic colitis has exceeded those of Crohn’s disease and ulcerative colitis among elderly persons. For example, in a recent Danish nationwide cohort study, the incidence of microscopic colitis (mean age at the time of diagnosis: 65 years) was 24.3 per 100,000 patient-years in 2016 vs. 18.6 for ulcerative colitis and 9.1 for Crohn’s disease per 100,000 patient-years in 2013 (Lophaven et al., 2004,; Weimers et al., 2020).

In general, the incidence of microscopic colitis has increased over time (Tong et al., 2015), Various factors, such as improved recognition of this disorder among gastroenterologists and pathologists, as well as varying presence of risk factors, may influence regional and temporal differences of the incidence. Thus, in a recent Danish nationwide cohort study, the incidence of microscopic colitis was found to have increased 10-fold, from 2.3 in 2001 to 24.3 cases per 100,000 patient-years in 2016 (Weimers et al., 2020). Similarly, a regional Swiss study described a statistically significant increase in incidence from 0.36 per 100,000 patient-years in 1994–1997 to 6.85 in 2017, with the mean age at diagnosis being 63 years (Maye et al., 2021). A similar trend was observed in North America (Gentile et al., 2014) with subsequent stabilization in incidence (Tome et al., 2022b).

A number of factors are linked to an increased risk of microscopic colitis, including a positive association to a broad spectrum of autoimmune diseases (Fedor et al., 2021), for example, celiac disease, type 1 diabetes mellitus, rheumatoid arthritis, polyarthritis, and thyroiditis (Wildt et al., 2021; Fernandez-Banares et al., 2013). Bile acid malabsorption is also associated ( Fernandez-Bañares et al., 2001).

Smoking status should be carefully reviewed as cigarette smoking is another risk factor associated with microscopic colitis (Jaruvongvanich et al., 2019). The risk appears to be up to five times higher in current smokers, with the disease onset occurring a minimum 10 years earlier compared to nonsmokers (Jaruvongvanich et al., 2019).

A variety of environmental factors, including a wide range of drugs, have been associated with the pathophysiology of microscopic colitis; however, with variable evidence suggesting causality (Zylberberg et al., 2021; Morgan et al., 2020). Thus, a certain cause–effect relationship between drug exposure and microscopic colitis has been described for proton pump inhibitors (PPIs), nonsteroidal anti-inflammatory drugs, or selective serotonin reuptake inhibitors (Verhaegh et al., 2016) both for current and recent use (Verhaegh et al., 2016; Bonderup et al., 2018). However, when using diarrheal controls instead of healthy controls, the association with several of these medications lessens or resolves (Zylberberg et al., 2021). The underlying mechanisms are not yet clarified, and these drugs could merely be triggers but not causative of inflammation in predisposed individuals. Regarding other medications claimed to be involved in the pathophysiology of microscopic colitis (Hamdeh et al., 2021), but with weaker associations, it should, however, be kept in mind that diarrhea is a common adverse event of multiple drugs appearing with a wide range of causes (Lucendo, 2017).

Finally, a nationwide Swedish case–control study found that the prevalence of previous gastrointestinal (GI) infection was significantly higher in microscopic colitis than in controls (Khalili et al., 2021). Another recent case–control study suggested that patients with microscopic colitis are less likely to be obese compared to those in diarrhea control groups (Sandler et al., 2022). These observations point toward the involvement of gut microbiota or hormonal effects of obesity.

## Etiopathology

**Figure 2.**
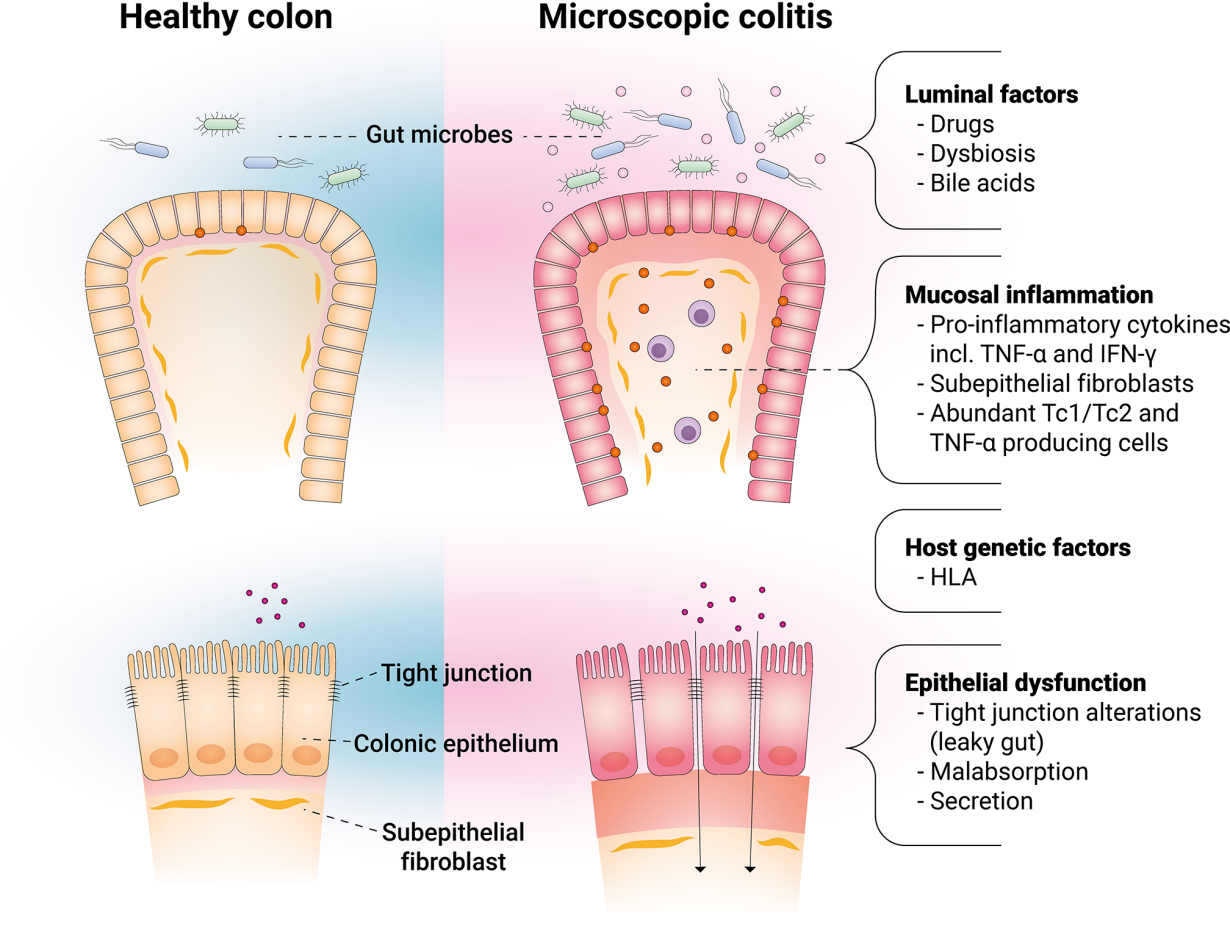
Main factors involved in the pathophysiology of microscopic colitis.

The pathogenesis of microscopic colitis is still poorly elucidated, but it is likely a result of dysbalanced immune response involving epithelial dysfunction (Barmeyer et al., 2017), collagen metabolism, secretory diarrhea (Escudero-Hernández et al., 2020), and microbiota (Khalili et al., 2021; Aagaard et al., 2021; Figure 2), combined with the risk factors mentioned above in genetically predisposed individuals (Ianiro et al., 2012). This complex area has previously been extensively covered by others (Zabana et al., 2022; Liu and Chen, 2022; Miehlke et al., 2019), and we therefore focused on new data in the fields of microbiota, genetic susceptibility, and SARS-CoV2.

### Alteration of microbiota

The bacterial flora in the colon is an important luminal factor that directly or indirectly interacts with colonic epithelium, and thus, its alteration might contribute to the pathogenesis of microscopic colitis. Although microscopic colitis is considered as a noninfectious colitis, recent advances in sequencing analysis have demonstrated an alteration of intestinal bacterial composition, referred to as dysbiosis. Thus, a recent sequencing study showed microbiota from microscopic colitis to be significantly less diverse and compositionally distinct from healthy controls due to depletion of members of *Clostridiales*; enriched for *Prevotella* and more likely dominated by this genus (Hertz et al., 2022). Two recent metagenomic studies revealed a lowered intestinal bacterial diversity and a reduction in the abundance of several genera in microscopic colitis, including *Akkermansia* and *Ruminococcus* (Carstens et al., 2019; van Hemert et al., 2018). The de-enrichment of *Akkermansia muciniphila* was additionally observed in a study using PCR (Fischer et al., 2015). *A. muciniphila* adheres to the intestinal epithelium and strengthens enterocyte monolayer integrity in vitro, suggesting that a reduction may cause intestinal barrier dysfunction (Reunanen et al., 2015). More recently, a team of researchers found a higher long-term risk of developing microscopic colitis in patients whose stool carried *Campylobacter concisus* (Aagaard et al., 2021; Nielsen et al., 2020). *Campylobacter concisus* is a commensal of the human oral microbiota, which occasionally may be isolated from stool samples. *C. concisus* is associated with epithelial sodium channel dysfunction and claudin-8-dependent barrier dysfunction (Nattramilarasu et al., 2020), suggesting their involvement in the pathogenesis of microscopic colitis. Notably, the intake of PPI and smoking influences bacterial flora (Fujimori, 2015; Shanahan et al., 2018), especially the former may especially increase the abundance of oral microbes, such as *C. concisus* (Schlenker and Surawicz, 2009), suggesting that dysbiosis may be the mechanism by which these factors cause microscopic colitis. Meanwhile, diarrhea itself might also change the bacterial composition (Li et al., 2021). Therefore, it remains unclear whether the dysbiosis is causal or secondary to microscopic colitis. Nevertheless, an altered intestinal microbiota composition is driven toward the composition of healthy controls once patients are in remission (Rindom Krogsgaard et al., 2019).

### Genetic susceptibility

A genetic component is of importance as well, and a recent genetic immunochip study with 4299 controls reported an association between the human leukocyte antigen (HLA) ancestral 8.1 haplotype and well-established collagenous colitis (314 patients) (Westerlind et al., 2017) but not with lymphocytic colitis (122 patients) (Westerlind et al., 2016). Furthermore, a significant genetic overlap was observed between ulcerative colitis and Crohn’s disease when comparing disease-associated single-nucleotide polymorphisms (SNPs) (Westerlind et al., 2017).

An array-based genetic association study on a cohort of 804 patients with collagenous colitis and more than 27,000 controls aimed to investigate a common genetic basis between collagenous colitis and Crohn’s disease, ulcerative colitis, or celiac disease (Stahl et al., 2020). In this detailed study, DNA was obtained from formalin-fixed, paraffin-embedded samples of colonic biopsies collected in routine colonoscopies. Here, an independent risk and protective HLA loci were implicated in the risk of collagenous colitis (Stahl et al., 2020). In this context, the HLA-DQ haplotype has long been known to be predisposed toward celiac disease and to be associated with microscopic colitis (Fine et al., 2000). The findings supported the role of HLA class I- and II-related mechanisms and identified potential non-HLA alleles linked to the pathogenesis of collagenous colitis (Stahl et al., 2020). Moreover, a cross-phenotype analysis identified a complex pattern of polygenic pleiotropy between collagenous colitis and other diseases, including celiac disease and IBD (Stahl et al., 2020).

Another team of researchers investigated phenotypic and genetic associations with microscopic colitis using a UK biobank with 483 white Europeans between 40 and 69 years of age at recruitment; an age span that covers the peak age for onset of microscopic colitis (Loreau et al., 2019; Sudlow et al., 2015). The team reported subsequent downstream analyses of genome-wide association studies (GWAS) (Green et al., 2019). This study stratified the data by drug use and employed GWAS to identify pharmacogenetic associations. Subsequently, a genetic risk score for IBD was calculated to quantify a genetic overlap with microscopic colitis (Green et al., 2019). Significant phenotypic associations with the use of PPIs (but with no other pharmacological risk factors), smoking status, and celiac disease were reported (Green et al., 2019). This group confirmed the aforementioned, recently reported association with the major histocompatibility complex ancestral 8.1. haplotype (Westerlind et al., 2017). By calculating risk scores, the researchers also reported suggestive evidence of a shared genetic risk with Crohn’s disease, but not with ulcerative colitis (Green et al., 2019). The UK biobank, however, depended on ICD-10 codes only to the first decimal place, which had limitations in terms of distinguishing lymphocytic from collagenous colitis, and also included the unrelated conditions of eosinophilic gastritis and colitis. Nevertheless, the research group was confident that their main GWAS result was not false positive due to the high minor allele frequency of the lead SNP and because it aligned closely with previous research. However, it was also acknowledged that having below 500 cases, the team was unable to detect the number of SNPs due to low odds ratio or minor allele frequency.

The aforementioned findings support the role of HLA class I- and II-related mechanisms and identified potential non-HLA alleles linked to the pathogenesis of collagenous colitis.

## SARS-CoV-2 infection and vaccination

The influence of SARS-CoV-2 infection on the GI system is well-established, with main symptoms being diarrhea, abdominal pain, loss of appetite, vomiting, nausea, and loss of taste (Jefremow and Neurath, 2021). However, onset of lymphocytic colitis has been observed rarely following SARS-CoV-2 infection with consistently elevated levels of fecal calprotectin and persistent diarrhea, even in the absence of fecal SARS-CoV-2 RNA (Nassar et al., 2019). Moreover, lymphocytic colitis has been reported immediately after the second SARS-CoV-2 mRNA vaccine dose (Chey et al., 2022). The authors additionally performed a review of the Centers for Disease Control and Prevention’s Vaccine Adverse Event Reporting System and revealed further five mRNA vaccine-related cases of microscopic colitis following both Pfizer-BioNTech and Moderna inoculations (three individuals developed severe diarrhea shortly after the second mRNA vaccine dose, whereas two individuals developed diarrhea approximately 1 month after receiving their second dose) (Chey et al., 2022). All diagnoses were confirmed via colonoscopy with biopsy. No cases of microscopic colitis have, however, been reported in patients receiving the Janssen single-dose vaccine. Although the number of cases reported is low compared to the huge number of vaccine doses administered, the observation is interesting and suggests that providers should consider microscopic colitis among the differential diagnoses of patients with persistent diarrhea following SARS-CoV-2 vaccination.

## Checkpoint inhibitors

With an increased use of immune checkpoint inhibitors (ICIs), that is neutralizing antibodies targeting the immune checkpoints T-lymphocyte-associated protein 4 (CTLA-4) or programmed cell death protein 1 (PD-1)/programmed cell death ligand-1 (PD-L1) for various malignancies, both lymphocytic colitis and collagenous colitis have been reported following therapy with these drugs (Rampersad et al., 2021; Choi et al., 2019; Isidro et al., 2021; Pai et al., 2021; Zou et al., 2020), although checkpoint inhibitor-induced (autoimmune) enteritis and colitis are far more frequent intestinal complications (Vaziri et al., 2022; Dougan et al., 2021). Accordingly, in case of diarrhea among patients presently on or previously treated with ICIs, the patients should always undergo a full colonoscopy as immune-mediated colitis is a frequent adverse event. Even in the absence of any endoscopic abnormalities, random biopsy samples should be obtained in order to assess the possibility of an underlying ICI-induced microscopic colitis (Dougan et al., 2021). Although budesonide may be successful for ICI-induced microscopic colitis, thus providing a compelling rationale for endoscopic identification (Hughes et al., 2019), it has been highlighted that microscopic colitis induced by ICIs may have a more aggravated, and even fatal, disease course (Fredrick et al., 2022). Such cases may require more potent immunosuppressive therapeutic regimens with biologics at an early point and a greater need for hospitalization than observed in a control group of non-ICI-induced microscopic colitis as well as traditional microscopic colitis without a history of cancer (Choi et al., 2019; Favara et al., 2020). In case of prescribing ICIs in patients with preexisting microscopic colitis, a close follow-up is recommended (Fredrick et al., 2021).

## Clinical presentation

One must always consider microscopic colitis in the event of unexplained intermittent symptoms of nonbloody, watery diarrhea. Patients often present additional symptoms, including urgency, fecal incontinence, abdominal discomfort, weight loss, arthralgias, and fatigue, impairing their quality of life. (Pardi, 2017 ). The onset is usually insidious, although sudden onset occurs in approximately 25% of patients (Olesen et al., 2021).

Given a severely affected quality of life due to a high overall symptom burden, diagnostic differentiation of microscopic colitis from other conditions of the GI tract is crucial. As symptoms are typically nonspecific, many patients meet the diagnostic criteria for various other conditions like irritable bowel syndrome, functional diarrhea, celiac disease, bile acid diarrhea, lactose malabsorption, and small intestinal bacterial overgrowth. Accordingly, these diagnoses all have to be considered should a patient with microscopic colitis not respond to standard therapy (Nimri et al., 2021; Poon et al., 2021; Misselwitz et al., 2019; Shiha et al., 2020).

## Diagnosis and assessment

The diagnosis of microscopic colitis is based on a full colonoscopy with histopathological assessment of multiple random biopsies obtained from the entire colon despite endoscopic absence of any macroscopic abnormalities. In a recent systematic review aimed at determining the optimal sites and minimum number of colonic biopsies required to diagnose microscopic colitis from published studies, it was concluded that a minimum of six biopsies should be obtained in total (three from the ascending and three from the descending colon) (Malik et al., 2022). The histological findings of microscopic colitis are, however, not uniform throughout the colon as the changes are more prominent in the proximal part of colon. Therefore, it is recommended that biopsies from the rectum be avoided as the chance of missing the diagnosis is highest here (Tanaka et al., 1992). The minimal required histopathological criteria for the subtypes of microscopic colitis seem to be achieved in >90% of the left-sided biopsies (rectum excluded), demonstrating a pancolitis in most patients with microscopic colitis (Fiehn et al., 2021). In this context, it has been revealed that a flexible sigmoidoscopy is often inadequate and may miss the diagnosis in >20% of all patients if biopsies are not obtained above the rectosigmoid colon (Chapman et al., 2015).

**Figure 3.**
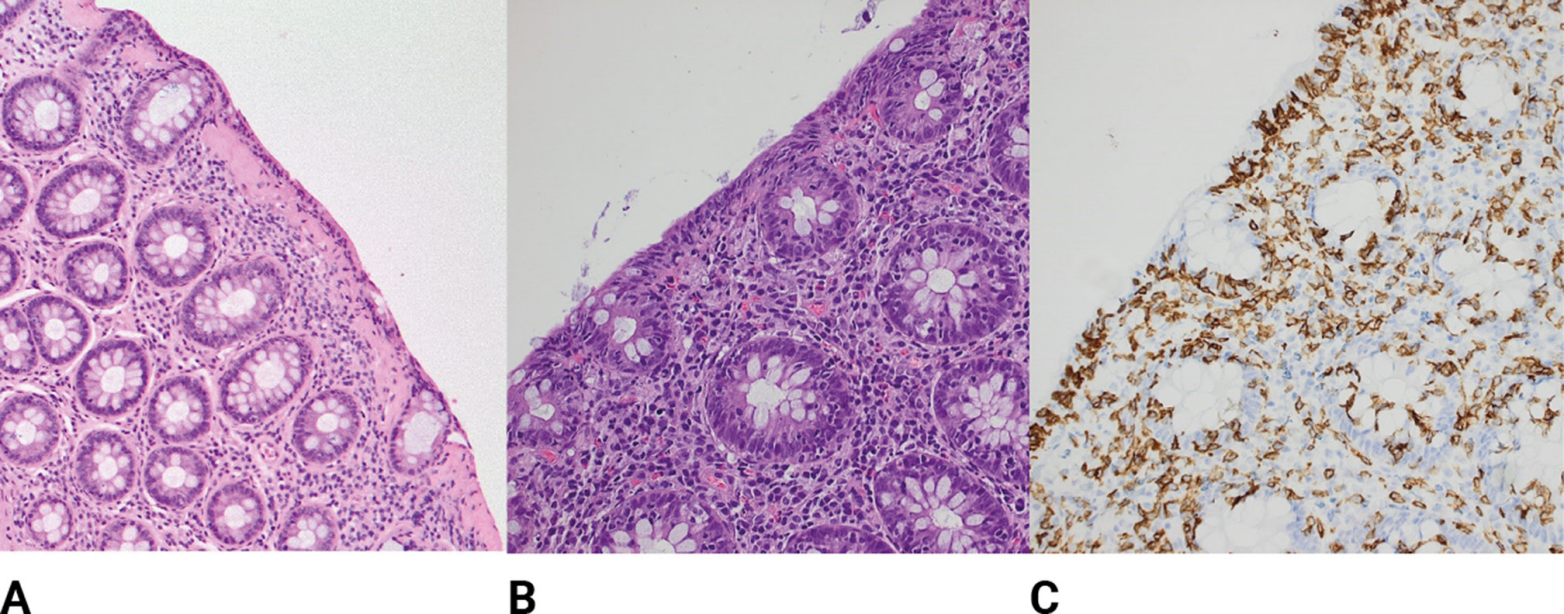
Histological findings of microscopic colitis. (**A**) Typical colonic biopsy from a patient with collagenous colitis with a subepithelial collagenous band of more than 10 μM. The surface epithelium is flattened, and mucin depleted, and a mixed inflammatory infiltrate is present in the lamia propria. H&E ×20. (**B**) Typical colonic biopsy from a patient with lymphocytic colitis with 20 or more intraepithelial lymphocytes per 100 surface epithelial cells. A mixed inflammatory infiltrate is present in the lamia propria. H&E ×20. (**C**) Lymphocytic colitis, immunohistochemistry stain for CD3 high-lighting lymphocytic infiltration of the epithelium.

When examining tissue specimens, the upper limit of the width of the normal collagen band in the subepithelial layer is approximately 5 µm. A collagen band >10 μM in width combined with an increased mixed inflammatory infiltrate in lamina propria defines collagenous colitis (Figure 3; Langner et al., 2015). The pathognomonic finding on biopsies in lymphocytic colitis is an intraepithelial lymphocytosis, defined as 20 or more intraepithelial lymphocytes (IEL) per 100 surface epithelial cells with a mixed infiltrate of acute and chronic inflammatory cells present in the lamina propria and a normal collagenous band (≤5 μM), typically without crypt distortion (Figure 3; Langner et al., 2015). However, mixed cases exist where histological changes of collagenous colitis are observed in some colonic segments, whereas other segments show changes of lymphocytic colitis (Fiehn et al., 2021; Engel et al., 2021). Moreover, some patients will change from one subtype to the other over time (Sonnenberg and Genta, 2022). Therefore, it is uncertain whether the two histological forms are really different entities or whether they could be different stages of a single pathological process, a phase of the process, or an individual response to different triggers. A recent analysis confirmed a synchronous occurrence of microscopic colitis and IBD and transitions between the two diagnoses (Sonnenberg and Genta, 2022). Furthermore, in addition to the widely accepted histologic criteria (Table 1), a broader histological definition of microscopic colitis with less prominent abnormalities, termed ‘incomplete microscopic colitis’ or ‘microscopic colitis not otherwise specified,’ has been proposed (Langner et al., 2015). This terminology is used to describe the subgroup of patients presenting with typical symptoms with either an abnormal collagenous layer or increased IELs that, however, are short of fulfilling the pathognomonic signs mentioned above (Langner et al., 2015).

**Table 1.** Key histological findings in microscopic colitis: differences between collagenous and lymphocytic colitis.

Parameter	Collagenous colitis	Lymphocytic colitis
Intraepithelial lymphocytes	Normal or increased number	>20 per 100 epithelial cells
Surface epithelium	Marked change (flattening, detachment)	Slight change (vacuolization, flattening, mucin depletion)
Subepithelial collagenous band	Thickening (≥10 μm)	Normal or slightly increased (<10 μm)
Inflammatory infiltrate in the lamina propria	Increased infiltrate (lymphocytes and plasma cells) with homogenous distribution throughout the colon	
Crypt architecture	Little or no distortion	
Inflammatory bowel disease-type focal changes	Occasional cryptitis and Paneth cell metaplasia	

## Treatment

In routine clinical practice, there is no difference in the treatment of lymphocytic colitis and collagenous colitis, as demonstrated by the approach of current European and American guidelines (Miehlke et al., 2021; Pardi et al., 2016; Nguyen et al., 2016). No significant differences exist in treatment response with regard to commonly used medications between younger (i.e., ≤50 years) and older (i.e., >50 years) patients with microscopic colitis (Sonnenberg and Genta, 2022 ; Kamboj et al., 2022).

The primary treatment goal is to achieve a clinical remission of microscopic colitis. In the frequent cases of a relapsing disease course, secondary goals are to maintain remission sufficiently, and in this way to improve the patient’s quality of life. It is unknown whether histological remission is an important goal, so in patients who respond clinically, repeat biopsies are not recommended.

The first step in management is to consider elimination of exacerbating factors; that is, to encourage smoking cessation and withdraw any culprit medications, that is, drugs with a suspected chronological relationship between drug introduction and onset of diarrhea. Although discontinuation of the offending drug leads to disease improvement in the majority of cases, (Hamdeh et al., 2021), there is no clear evidence yet of how it might predictably alter the disease course.

### First-line therapy

#### Budesonide

Budesonide, a synthetic, locally acting glucocorticoid with topical effects in the GI tract, is the mainstay for induction and maintenance of remission in this disorder, based on randomized placebo-controlled trials and meta-analyses. (Chande et al., 2017; Kafil et al., 2017; Sebastian et al., 2019).

Budesonide is recommended as first-line therapy according to both American Gastroenterological Association guidelines and the European Microscopic Colitis Group statements (Miehlke et al., 2021; Pardi et al., 2016; Nguyen et al., 2016). This second-generation glucocorticoid allows local selective therapy of the GI tract by high-affinity binding to the intracellular glucocorticoid receptor (Miehlke et al., 2018a). Moreover, extensive (90%) first-pass metabolism within the liver and the mucosa of the small intestine reduce the systemic availability (Miehlke et al., 2018a), which diminishes the risk of side effects and obviates the need for dose tapering during induction therapy. Thus, no cases of adrenal insufficiency have been reported in prospective clinical studies when discontinuing a daily dosage of 9 mg for 6–8 weeks without tapering.

Budesonide administered orally has been assessed for induction of remission in four randomized-controlled trials of collagenous colitis (Kafil et al., 2017) and three of lymphocytic colitis (Chande et al., 2017). The proportions of patients in clinical remission after 6–8 weeks of treatment ranged from 72 to 91% of those with lymphocytic colitis, and from 73 to 100% of those with collagenous colitis. The frequency of watery diarrhea decreased quickly, usually within 2 weeks of treatment initiation (Miehlke et al., 2018b). Accordingly, first-line treatment of active microscopic colitis is a dosage of 9 mg budesonide once daily for 8 weeks (Miehlke et al., 2018b). Budesonide has even shown efficacy in the management of incomplete microscopic colitis (Münch et al., 2021).

As recurrence is a frequent phenomenon after a short-term budesonide therapy (Sebastian et al., 2019), maintenance therapy may be considered. Randomized trials of maintenance therapy, in which 4.5 or 6 mg of budesonide was administered per day for 6–12 months after remission, revealed that remission was sustained in 60–75% of patients, and that treatment was well-tolerated without any specific safety issues (Bonderup et al., 2009; Miehlke et al., 2008). Nevertheless, in one study, around 80% of patients were reported to have relapsed after withdrawal of long-term budesonide therapy (Münch et al., 2016). In the event of relapse, the patient should accordingly be reassessed and retreated, aiming to taper to the lowest possible dose of budesonide, for example, 3 mg alternate days, but the duration of any maintenance therapy is yet unknown. Moreover, as long-term use of glucocorticoids is controversial, calcium and vitamin D supplementation should be recommended to the subgroup of patients requiring budesonide maintenance. Physicians often consider monitoring bone mineral density in patients treated with budesonide maintenance therapy, although budesonide may have less impact on bone density than a traditional glucocorticoid like prednisolone (Schoon et al., 2005), and a recent study reported a low risk of metabolic bone disease (and other steroid-related side effects) that was not significantly different from patients not treated with budesonide (Tome et al., 2022a).

### Second-line therapy

In case patients with microscopic colitis fail to respond to budesonide to induce and maintain clinical remission, and in those who develop significant side effects, additional therapies, such as loperamide, bile acid sequestrants, bismuth subsalicylate, thiopurines, and biologicals, may be considered.

#### Loperamide

Even though there is not enough evidence to recommend loperamide, as this antidiarrheal medication has never been systematically studied in relation to microscopic colitis, loperamide might, however, be an option as symptomatic therapy in patients with urgency as well as mild microscopic colitis. However, in one large case series, antidiarrheal therapy by itself was able to induce remission in only a small fraction of patients (Pardi et al., 2002). Thus, loperamide may have a better role as combination therapy when symptoms do not respond completely to a medication such as budesonide.

#### Bile acid sequestrants

Cholestyramine (a bile acid-binding resin used for diarrhea due to bile acid malabsorption) may be helpful, especially in the substantial number of patients with microscopic colitis and coexisting bile acid malabsorption (Fernandez-Bañares et al., 2001; Ung et al., 2000). Moreover, bile acid sequestrant therapy may be effective in decreasing budesonide dependence in half the patients with microscopic colitis (Northcutt et al., 2022). Thus, cholestyramine, at a dose of 4 g 3–4 times per day (starting with a dose of 8 g per day and increased slowly over time depending on clinical response), may be of benefit in such cases or as an option following budesonide induction (Northcutt et al., 2022). Furthermore, since the selenium homocholic acid taurine abdominal retention test is not available in all countries, and since the antidiarrheal effect of cholestyramine is nonspecific, it may be prudent to try this medication prior to escalating therapy with an immunomodulatory or biological agent. A recent study suggested that a serum marker of bile acid malabsorption, 7-α-hydroxy-4-cholesten-3-one, may predict response to colesevelam in microscopic colitis (Saha et al., 2020). Colesevelam is a bile acid sequestrant shown to be efficacious in bile acid malabsorption in Crohn’s disease (Beigel et al., 2014).

#### Bismuth subsalicylate

Bismuth subsalicylate, having antisecretory and anti-inflammatory properties, reduces diarrhea in microscopic colitis. However, the antidiarrheal mechanism of action still has to be determined (Ericsson et al., 1990).

Bismuth subsalicylate (nine tablets [262 mg each] per day in three divided doses) performed better than placebo in a randomized trial (Fine et al., 1999). In another study, the clinical response and recurrence rates in 94 patients (median age of 69 years; 91% females, 52% with lymphocytic colitis) treated with this drug were assessed (Tariq et al., 2017). The daily dose of bismuth subsalicylate was six tablets in 12 patients, eight tablets in 20 patients, and nine tablets in 62 patients. Overall, 57% patients showed complete response, 21% had partial response, and 21% had no response. In this context, it is noteworthy that long-term use of this drug is associated with the risk of neurotoxicity (Borbinha et al., 2019). Nevertheless, to determine the true benefit of bismuth subsalicylate, well-powered, prospective, placebo-controlled studies stratified by disease severity and subtypes need to be performed.

#### Thiopurines

When symptoms are truly refractory and considerably impact the quality of life, immunomodulating therapy with thiopurines (i.e., azathioprine [2–2.5 mg/kg per day] or mercaptopurine [1–1.5 mg/kg per day]) for maintenance of clinical remission may be initiated, but as the onset of action is delayed (10–12 weeks), thiopurines are not indicated as induction therapy (Nielsen et al., 2013).

In a cohort study of budesonide-refractory, -dependent, or -intolerant patients (Cotter et al., 2017), thiopurines were used in 49 patients for a median duration of 4 months (IQR 1.5–15.0). Complete or partial responses were observed in 43% of patients with collagenous colitis and 22% with lymphocytic colitis. In a multicenter and retrospective case series, 13 (28%) of 46 patients treated with azathioprine achieved and maintained clinical remission for up to 57 months, whereas 31 (67%) patients developed intolerance causing treatment cessation (Münch et al., 2013). Thirteen of these patients were subsequently switched to mercaptopurine, among whom six (46%) regained clinical remission. Thus, overall, 19 (41%) patients responded to the treatment. Although it is still not clear as to how long thiopurines should be administered in case of a beneficial response, it is recommended that they should be administered for a minimum of 1 year, and perhaps even longer.

#### Biologicals

In recent years, TNF inhibitors (Nielsen and Ainsworth, 2013) (infliximab and adalimumab) have been introduced for the management of microscopic colitis (Cotter et al., 2017; Münch et al., 2012; Anderson and Makins, 2016; Daferera et al., 2019; Pola et al., 2013), together with the α_4_β_7_ anti-integrin, vedolizumab (Hollier et al., 2022; Shipley et al., 2022; Abu-Sbeih et al., 2018; Rivière et al., 2019) and recently the anti-IL-12/IL-23 antibody, ustekinumab (Abughazaleh et al., 2019). A meta-analysis of 11 articles (four with infliximab and adalimumab, two with adalimumab, and five with vedolizumab), including 75 patients with severe microscopic colitis, showed a response rate of 77% at 3–6 weeks and 67% at 12–16 weeks, and 55% were able to achieve remission at 12–16 weeks (Taneja et al., 2021). As only one case study exists for ustekinumab, a response rate must await further studies. Moreover, a recent multicenter cohort study was the first to show that about half of the patients treated with TNF inhibitors for microscopic colitis achieved clinical remission following budesonide failure (Boivineau et al., 2022).

#### Therapy with other anti-inflammatory or antidiarrheal drugs

Additional agents may be trialed as alternatives. However, some of these drugs are less well-studied, and they were not all recommended by the latest European (2021) guidelines for the management of microscopic colitis (Miehlke et al., 2021).

#### Small molecules

So far it is unknown whether the new small molecules like selective second-generation Janus kinase inhibitors (acting as cytokine inhibitors) (Soendergaard et al., 2018) or sphingosine-1-phosphate receptor modulators that ‘trap’ lymphocytes in the lymph nodes (Nielsen et al., 2017) could be an option in microscopic colitis (e.g., the latter of potential relevance for lymphocytic colitis).

#### Prednisolone

Prednisolone is a glucocorticoid often used for classical IBD. However, current research, including only one placebo-controlled trial with prednisolone (Munck et al., 2003), a population-based cohort study, (Gentile et al., 2013), and a meta-analysis (Stewart et al., 2011), suggests that prednisolone is less effective than budesonide for both collagenous and lymphocytic colitis. Accordingly, this drug is not recommended for use in microscopic colitis (Miehlke et al., 2021).

##### Methotrexate

One study has indicated an effect of methotrexate in microscopic colitis (Cotter et al., 2017). However, 75% of the patients in the study received concomitant budesonide, which limits the conclusions to be drawn about methotrexate as monotherapy. Meanwhile, other studies have described a relative ineffectiveness of methotrexate in microscopic colitis (Münch et al., 2013; Münch et al., 2012). Therefore, this drug is not recommended for microscopic colitis management.

### Mesalazine

Mesalazine administered in a dose of 3 g per day for 8 weeks was not superior to placebo in patients with collagenous colitis (Miehlke et al., 2014) or lymphocytic colitis (Miehlke et al., 2018b). Based on these data and supported by real-life experience for induction of remission in both collagenous and lymphocytic colitis (no studies for the maintenance of remission are available) (Bohr et al., 1996), 5-aminosalicylic acid is not recommended as a therapeutic option.

## Surgery

Data on the role of surgery (i.e., subtotal colectomy, diverting ileostomy, or an ileal pouch-anal anastomosis) are limited to isolated case reports and should be regarded as the ultimate treatment option to be reserved for patients with a disease course refractory to all other medical therapies or when effective medication cannot be tolerated (Bislenghi et al., 2022 ; Münch et al., 2005; Järnerot et al., 1996).

## Prognosis

Although several studies have demonstrated an association between Crohn’s disease and ulcerative colitis and the risk of colorectal cancer (Beaugerie and Itzkowitz, 2015; Olén et al., 2020b; Olén et al., 2020a), previous studies focusing on the risk of colorectal cancer in microscopic colitis have not revealed such an association (Loreau et al., 2019; Levy et al., 2019; Borsotti et al., 2021). A systemic review and meta-analysis of 12 studies comprising 50,795 patients with microscopic colitis even found a lower risk of colonic adenomas and colorectal cancer (Liu et al., 2021). Nevertheless, a Swedish nationwide, population-based cohort study recently examined the association between microscopic colitis and overall risk of cancer as well as of some important cancer subtypes (Bergman et al., 2020). In this first large-scale study, an 8% elevated risk of overall cancer was revealed in individuals with microscopic colitis compared with reference individuals. (Bergman et al., 2020). Specifically, an increased risk of lymphoma and lung cancer was discovered in patients with microscopic colitis, even when performing a sensitivity analysis to account for the influence of immunomodulatory drugs on the risk for lymphoma, although a confounder for lung cancer may probably be due to a higher prevalence of smoking among patients with microscopic colitis (Bergman et al., 2020). However, there were no associations found with breast, bladder, or colorectal cancers or with other GI malignancies (Bergman et al., 2020). Moreover, the study demonstrated that the risk of cancer did not increase with the duration of disease (Bergman et al., 2020). Consequently, repeated colonoscopy with biopsies is seemingly not required in microscopic colitis, unless a patient’s progress mandates further evaluation or suggests a possible alternative diagnosis.

In a recent study, the clinical course over time in a cohort of 318 patients with microscopic colitis and a mean age of 64 years with complete 1-year follow-up revealed that 49% had a chronic active or relapsing disease course; 40% achieved sustained remission after treatment, and 11% had a quiescent course (Verhaegh et al., 2021). In general, symptoms and quality of life improved after 3 months of follow-up. A relapsing or chronic active disease course was associated with considerably more symptoms and an impaired quality of life after 1 year (Verhaegh et al., 2021). Previously, another study evaluating the natural history of microscopic colitis with a median follow-up of 8 years revealed that 75% of patients achieved prolonged remission without therapy for more than a year (Fernández-Bañares et al., 2016). Remarkably, 93% of patients achieving remission spontaneously went on to have prolonged remission, whereas only 61% of those who achieved drug-induced remission had prolonged remission for more than a year (Fernández-Bañares et al., 2016).

## Disease monitoring and long-term assessment

Patients should be offered a regular follow-up until the symptoms resolve, and at least every 12 months thereafter with the goal of maintaining control over the chronic disorder and ensuring easy access to treatment at a specialized outpatient clinic in case of flaring up of the disease. In case patients require long-standing glucocorticoid treatment to control their disease, supplementation of calcium and vitamin D is recommended, and these individuals should be monitored regarding osteoporosis. Moreover, appropriate selection of patients for colonoscopy is essential for reducing healthcare expenses as this procedure might be overused if performed on patients with well-established microscopic colitis who do not experience a significant change in their symptoms (Kim et al., 2021).

## Current controversies and future directions

Despite microscopic colitis becoming increasingly recognized as a common cause of diarrhea, especially among the elderly, awareness needs to be raised further among healthcare providers in countries with a low reported incidence.

On the basic science level, a better understanding of the underlying etiopathology of microscopic colitis is clearly needed. One of the most critical issues is to identify the causes and triggers that lead to the development of this disorder. The key observation that fecal stream diversion makes the inflammation almost disappear indicates the importance of luminal factors to maintain the inflammatory process (Järnerot et al., 1996; Järnerot et al., 1995). Recent metagenomic studies have identified intestinal microbiota linked to microscopic colitis (Carstens et al., 2019; van Hemert et al., 2018). Therefore, revealing the role of the gut microbiota and manipulation hereof, but also of cytokine imbalances and/or gene signatures, might open opportunities for devising new rational therapeutic strategies that can prevent disease development and flare-ups or perhaps even provide a cure. In this way, a combination of omics and bioinformatics could likely contribute to the efforts of revealing the origin of this disorder and might even assist in decision-making of selecting the right treatment for the individual patient.

Available treatments for microscopic colitis have limitations, and new treatment options are needed. Until now, the management of microscopic colitis has aimed at resolving symptoms and improving patients’ quality of life in the short term. Oral budesonide has for years been the treatment of choice for induction and maintenance of remission in most patients. However, it is still unclear as to how long patients should be treated with maintenance therapy or if any prophylactic medication exists. Budesonide-refractory microscopic colitis remains an important clinical challenge. Medications other than those well-established in the treatment regimen of the more classical forms of IBD may be beneficial in reducing the risk of relapse and maintaining remission in the long term. Moreover, it is yet not clear if collagenous colitis and lymphocytic colitis should be treated as two different entities.

An unmet need is the development and validation of noninvasive biomarkers like fecal calprotectin (Batista et al., 2019) or mucosal and fecal neutrophil gelatinase-associated lipocalin (Bakke et al., 2021) as potential biomarkers for microscopic colitis to assess and predict clinical disease activity. Thus, a very recent study of 166 patients with microscopic colitis, yielding 234 independent instances of fecal calprotectin measurement during symptomatic relapse, showed elevated concentrations of fecal calprotectin to be associated with more severe symptoms (Sasson and Ananthakrishnan, 2022). Also required is a toolkit to define and measure disease severity as well as distinguish microscopic colitis from other functional or organic causes of watery diarrhea. Accordingly, until noninvasive testing is identified, all patients suspected of active microscopic colitis must be directly referred to colonoscopy with multiple biopsies obtained, followed by an extensive histopathological evaluation. This is the only way to make a definitive diagnosis.

**Figure 4.**
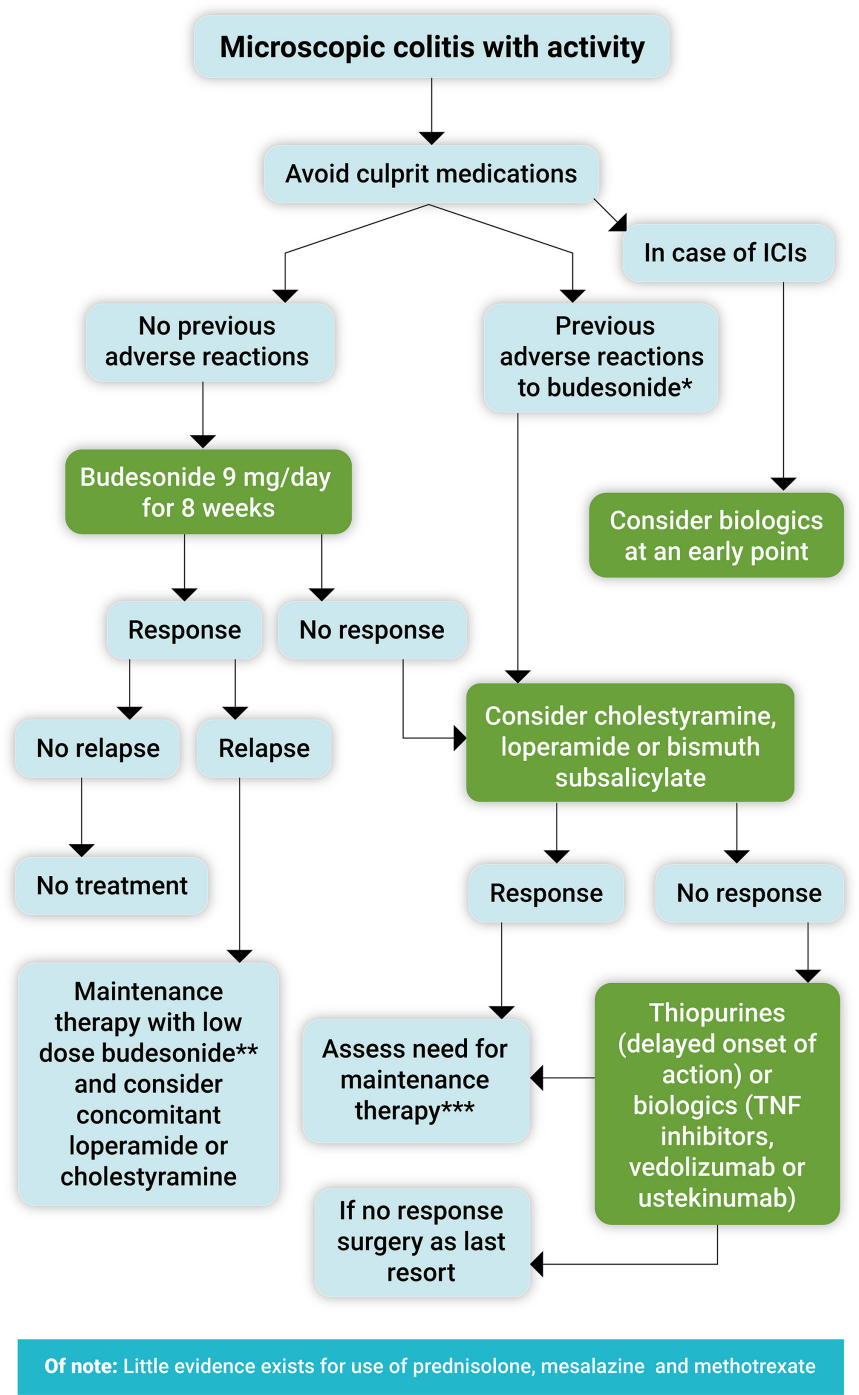
Proposed treatment algorithm for the clinical management of symptomatic microscopic colitis. Immune checkpoint inhibitors (ICIs). *Loperamide or bismuth subsalicylate in mild cases, cholestyramine mainly if there is associated bile acid malabsorption. **Use the lowest effective dose as maintenance treatment (3–6 mg/day or 3 mg every other day). ***Long-term use of bismuth subsalicylate is not recommended due to potential neurotoxicity. Note: celiac disease should each time be excluded at least with anti-tissue transglutaminase antibody levels, and bile acid diarrhea always kept in mind.

Based on the most up-to-date knowledge, a proposed treatment algorithm for the management of microscopic colitis is provided (Figure 4). However, the healthcare provider should first recommend that smokers quit and consider any drug-induced causes of diarrhea.

In conclusion, as flaring microscopic colitis can be a debilitating disorder with concerns related to urgency and fecal incontinence, the disease should always be considered in case of unexplained watery diarrhea. Thus, microscopic colitis deserves the same attention as the classical IBDs in the effort to bring flaring disease into sustained remission.
